# MRI-safe fiber electrodes for neuromodulation in the deep brain

**DOI:** 10.1093/nsr/nwag399

**Published:** 2026-06-30

**Authors:** Guo Tang, Xing Sheng

**Affiliations:** Department of Electronic Engineering, IDG/McGovern Institute for Brain Research, Tsinghua University, China; Department of Electronic Engineering, IDG/McGovern Institute for Brain Research, Tsinghua University, China

Electrical neuromodulation including deep brain stimulation (DBS) treats intractable neurological and psychiatric disorders such as Parkinson’s disease and autism spectrum disorder (ASD) [1–3]. Unraveling stimulation-evoked whole-brain circuits demands synchronized electrophysiology and multimodal magnetic resonance imaging (MRI) readout, a combination blocked by conventional implantable electrodes [4]. Clinically used platinum-iridium (PtIr) probes induce severe MRI artifacts via magnetic susceptibility mismatch and cause chronic brain inflammation due to extreme mechanical mismatch with soft neural tissue, leading to unstable long-term recording signals [1,5].

Previously developed carbon-based graphene fiber electrodes relieve partial imaging interference [6], yet they only operated below 9.4 T and did not integrate functional MRI (fMRI), diffusion-weighted imaging (DWI), and magnetic resonance spectroscopy (MRS) for deep-brain analysis [5–7]. Other soft polymer electrodes are limited to superficial cortical implantation and cannot support deep multimodal detection [4,8]. Furthermore, no existing device enables concurrent stimulation, electrophysiology, and multiparametric MRI at a high magnetic field of 11.7 T, restricting mechanistic research of deep neuromodulation [4,9].

**Figure 1.**
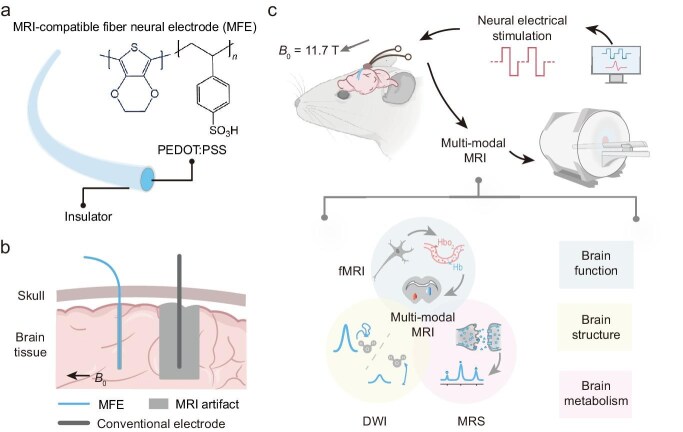
Multimodal investigation of neural modulation mechanisms with MFEs. (a) Schematic illustration of the MFE based on PEDOT:PSS. (b) Schematic illustration of the MRI artifacts induced by an MFE and a conventional MRI-incompatible neural electrode. (c) Schematic illustrating multimodal investigation of neuromodulation mechanisms through electrical stimulation synchronized with multimodal MRI, including fMRI, DWI, and MRS at 11.7 T. Reproduced from ref. [10] with permission.

In a recent study, Peng and co-workers report a structurally optimized MRI-compatible fiber neural electrode (MFE) based on a poly(3,4-ethylenedioxythiophene) polystyrene sulfonate (PEDOT:PSS) conductive polymer gel (Fig. 1a), which closely matches the magnetic susceptibility (−9.23 ppm) of brain tissue (−9.05 ppm) and delivers ultralow Young’s modulus (328 kPa) far superior to all prior MRI-compatible neural probes [10]. At an 11.7 T ultrahigh field, MFEs produce negligible signal loss and artifact pixels ∼10-fold smaller than PtIr counterparts across 20–80 μm diameters (Fig. 1b), with minimal long-term implantation inflammation and radiofrequency heating risk. Electrochemically, the porous fiber architecture endows MFEs with two orders-of-magnitude higher charge injection limit (14.3 mC cm^−2^) and far lower interfacial impedance, supporting high-fidelity single-unit recording and spatially precise localized stimulation.

Integrating MFEs into flexible chronic neural interfaces, the team realized simultaneous medial prefrontal cortex (mPFC) electrical stimulation, local field potential recording, and triple-modal MRI scanning in wild-type and MECP2-duplication autism model rats (Fig. 1c). Frequency-dependent whole-brain mapping revealed that high-frequency (130 Hz) mPFC stimulation reverses pathological functional and microstructural aberrations across autism-associated brain regions, alongside distinct metabolic shifts in glutamate and taurine.

In sum, the MFE platform removes the long-standing technical bottleneck separating electrophysiological neuromodulation and ultrahigh-field multimodal MRI, constructing a universal analytical toolkit for dissecting whole-brain circuit dynamics. Looking ahead, this soft fiber bioelectronics holds promising translational prospects for human DBS clinical practice [1]. Real-time intra-operative MRI navigation with artifact-free electrodes will allow precise target localization for ASD, depression, and movement disorder treatments, while chronic post-implant MRI follow-up can dynamically track long-term circuit remodeling induced by stimulation [2,7]. Nevertheless, several translational hurdles remain unresolved: scalable microarray fabrication for multiregion parallel recording, long-term biostability in human cerebrospinal fluid, and optimized packaging compatible with clinical 3 T/7 T MRI scanners. Further material modification and system miniaturization of PEDOT:PSS fiber electrodes will bridge the gap between rodent laboratory research and human clinical neuromodulation therapies, advancing personalized, mechanism-guided brain intervention [8,9].
